# 2.F. Skills building seminar: Health Data Pipelines: moving away from Excel to scalable, insightful and future-proof infostructure

**DOI:** 10.1093/eurpub/ckac129.081

**Published:** 2022-10-25

**Authors:** Organised by: EUPHA-DH, Chair persons: Stefan Buttigieg (EUPHA-DH), Juan Rachadell (Portugal)

## Abstract

Public Health Data Pipelines are critical in the implementation of scalable Digital Public Health projects as part of the reporting, evaluation and monitoring aspects of any public health intervention. The WHO Europe Programme of Work covering the period 2020 to 2025 puts an emphasis on “developing big-data capacity in surveillance, modelling and policy monitoring”. This workshop will provide an initial insight into the world of Health Data Pipelines and will give you the necessary ingredients to get closer to establishing processes and systems that provide you the right information at the right time and context.

**Key messages:**

• Data Linking is critical in the transition between legacy systems and data sets based on decades old technology to reliable and robust information infrastructure (infostructure).

• Kicking off the necessary internal discussions to consolidate data and processes into health data pipelines do not require advanced technical knowledge.

